# O2.7. CAN THEY HEAR IT? DO PATIENTS WITH AUDITORY VERBAL HALLUCINATIONS HAVE AUDITORY PROCESSING DEFICITS?

**DOI:** 10.1093/schbul/sby015.197

**Published:** 2018-04-01

**Authors:** Susan Rossell, Eric Tan, Nathan Wilson, Wei Lin Toh, Sean Carruthers, Philip Sumner, Matthew Hughes, Neil Thomas

**Affiliations:** 1 Swinburne University; 2 CMH, Swinburne University, Monash Alfred Psychiatry Research Centre, The Alfred Hospital and Monash University

## Abstract

**Background:**

It has been well established for over half a century that patients with a diagnosis of schizophrenia have auditory processing deficits. More recently, there has been a small body of work that has suggested patients with a history of auditory hallucinations have particularly pertinent auditory processing deficits, with the importance of pitch perception being noted in a single study. This current body of work has systematically investigated whether auditory perception, using a tone detection task, are related to auditory verbal hallucinations (AVH).

**Methods:**

Four studies will be presented each of which used a tone detection task that manipulated pitch, with the study 1 also have a tone duration manipulation.

**Results:**

Study 1 compared 15 AVH with 15 non-AVH patients with schizophrenia with 34 healthy controls, AVH patients demonstrated the greatest impairments on pitch and tone duration deficits compared to the other two groups. Study 2 compared 99 patients with schizophrenia (65 with a history of AVH and 34 without) with 95 healthy controls and confirmed pitch deficits in schizophrenia, specifically those with AVH. Study 3 established in 100 healthy controls that unusual experiences or auditory hallucination proneness were significantly correlated to pitch perception performance. Lastly, study 4 demonstrated that relatives of patients with schizophrenia also showed a significant correlation between usual experiences and pitch perception.

**Discussion:**

This research indicates auditory processing deficits are a core feature of AVH in schizophrenia, and potentially represent an endophenotype for AVH. The authors will discuss a potential cognitive model which explains the relationship between AVH and pitch perception. There are clear translational elements to this research, and we suggest there might be some utility in using auditory training as an intervention to reduce the impact of AVH.

